# Simultaneous Determination of Oxysterols, Cholesterol and 25-Hydroxy-Vitamin D3 in Human Plasma by LC-UV-MS

**DOI:** 10.1371/journal.pone.0123771

**Published:** 2015-04-13

**Authors:** Rohini Narayanaswamy, Vignesh Iyer, Prachi Khare, Mary Lou Bodziak, Darlene Badgett, Robert Zivadinov, Bianca Weinstock-Guttman, Todd C. Rideout, Murali Ramanathan, Richard W. Browne

**Affiliations:** 1 Department of Biotechnical and Clinical Laboratory Sciences, University at Buffalo, State University of New York, Buffalo, New York, United States of America; 2 Department of Exercise and Nutrition Sciences, University at Buffalo, State University of New York, Buffalo, New York, United States of America; 3 Department of Pharmaceutical Sciences, University at Buffalo, State University of New York, Buffalo, New York, United States of America; 4 Department of Neurology, University at Buffalo, State University of New York, Buffalo, New York, United States of America; Clermont Université, FRANCE

## Abstract

**Background:**

Oxysterols are promising biomarkers of neurodegenerative diseases that are linked with cholesterol and vitamin D metabolism. There is an unmet need for methods capable of sensitive, and simultaneous quantitation of multiple oxysterols, vitamin D and cholesterol pathway biomarkers.

**Methods:**

A method for simultaneous determination of 5 major oxysterols, 25-hydroxy vitamin D3 and cholesterol in human plasma was developed. Total oxysterols were prepared by room temperature saponification followed by solid phase extraction from plasma spiked with deuterated internal standards. Oxysterols were resolved by reverse phase HPLC using a methanol/water/0.1% formic acid gradient. Oxysterols and 25-hydroxy vitamin D3 were detected with atmospheric pressure chemical ionization mass spectrometry in positive ion mode; in-series photodiode array detection at 204nm was used for cholesterol. Method validation studies were performed. Oxysterol levels in 220 plasma samples from healthy control subjects, multiple sclerosis and other neurological disorders patients were quantitated.

**Results:**

Our method quantitated 5 oxysterols, cholesterol and 25-hydroxy vitamin D3 from 200 μL plasma in 35 minutes. Recoveries were >85% for all analytes and internal standards. The limits of detection were 3-10 ng/mL for oxysterols and 25-hydroxy vitamin D3 and 1 μg/mL for simultaneous detection of cholesterol. Analytical imprecision was <10 %CV for 24(S)-, 25-, 27-, 7α-hydroxycholesterol (HC) and cholesterol and ≤15 % for 7-keto-cholesterol. Multiple Sclerosis and other neurological disorder patients had lower 27-hydroxycholesterol levels compared to controls whereas 7α-hydroxycholesterol was lower specifically in Multiple Sclerosis.

**Conclusion:**

The method is suitable for measuring plasma oxysterols levels in human health and disease. Analysis of human plasma indicates that the oxysterol, bile acid precursors 7α-hydroxycholesterol and 27-hydroxycholesterol are lower in Multiple Sclerosis and may serve as potential biomarkers of disease.

## Introduction

There is increasing evidence that serum cholesterol and lipoprotein levels are associated with multiple sclerosis (MSC) disease progression [1, 2]. The mechanisms responsible for these disease associations are not known.

The associations between circulating cholesterol levels in MSC and other neurodegenerative diseases (OND) may potentially be mediated via the oxysterols and secosterols [3]. Oxysterols are enzymatic and autooxidation products of cholesterol with one or more oxygen containing functional groups including hydroxy, keto, hydroperoxy, and epoxy moieties attached at different positions on the ring structure or the side chain chain [4, 5]. Secosterols are compounds containing a steroid B-ring with a broken bond between C-9 and C-10. Vitamin D and its metabolites are the prototypical endogenous secosterols.

Oxysterols are intermediates in cholesterol metabolism and are important regulators of cholesterol homeostasis in the periphery and the brain. Cholesterol homeostasis in the brain is critically important for myelin integrity and function. The lipophilicity of free cholesterol limits its transport into and out of the brain. The brain therefore synthesizes the cholesterol it needs and excess cholesterol is converted to 24(*S*)-hydroxycholesterol (24HC) for excretion [6–8]. Evidence indicates that oxysterol levels are altered in neurodegenerative diseases including MSC [9–11], Alzheimer’s disease [12–14] and Parkinson’s disease [15]. Beyond neurodegenerative diseases there is evidence that oxysterols may play a role in certain cancers. In particular, 27HC may have a role in the link between hypercholesterolemia and breast cancer pathophysiology [16].

Cholesterol is biochemically connected to oxysterols and also to the endogenous vitamin D synthesis pathway via 7-dehydro cholesterol. Vitamin D metabolites (25-hydroxyvitamin D3 and 25-hydroxyvitamin D2) are the major circulating secosterol forms and are hydroxylated at C-25. Low vitamin D levels are associated with MSC susceptibility and disease progression [2, 17, 18] and with Alzheimer’s disease [19]. In MSC patients, vitamin D levels also are inter-dependent on cholesterol biomarkers [20–22].

Bioanalytical chromatography oxysterol analysis methods have focused on initial extraction of total sterols followed by separation of oxysterols from cholesterol prior to analysis [23]. Secosterols, predominantly vitamin D, are typically quantified separately from oxysterols. Importantly, recent studies in neurodegenerative diseases indicate that oxysterol and secosterol levels relative to the total cholesterol levels are more useful than absolute levels as biomarkers in these disease states [11, 24]. A method capable of simultaneous determination of total cholesterol, oxysterols and secosterols would be an efficient tool for the further investigation of these physiologically inter-connected biomarkers in neurodegenerative diseases.

Analysis of oxysterols and secosterols in biological matrices is complicated by four major factors: i) the low concentrations of oxysterols/secosterols in the presence of a large excess of cholesterol [25], ii) the potential for generation of oxysterol artifacts during sample preparation and analysis [23], iii) the presence of the majority of oxysterols as fatty acid esters *in vivo* [7, 26], and iv) the existence of isomeric forms (isobars) which have similar chromatographic and mass spectral properties [25].

We have developed a robust, parsimonious and effective liquid chromatography-mass spectrometry (LC-MS) technique capable of the resolving 12 oxysterol compounds, 25-hydroxyvitamin D3, 25-hydroxyvitamin D2, 3 cholesterol precursors and total cholesterol. We have validated 7 of these compounds for routine measurement in human plasma. The reverse-phase method entails a simple binary gradient with methanol-water containing 0.1% formic acid and dual detection using photodiode array in series with single quadrapole, atmospheric pressure chemical ionization (APCI) mass spectrometry (MS). We report here on the validation of this method according to Food and Drug Administration (FDA) guidelines for bioanalytical methods and demonstrate its utility in analyzing plasma samples for healthy controls (HC), MSC and OND subjects.

## Materials and Methods

The reagent and standards sources, preparation of calibrators, internal standards, quality control samples and the blank matrix preparation methods are described in the expanded description of experimental procedures within S1 File.

### Sample Preparation and Analysis

Our method involves alkaline hydrolysis of lipid esters followed by solid phase extraction prior to LC-MS analysis.

### Alkaline Hydrolysis/Saponification

For analysis 200 μL of sample (calibrators, QC or unknown plasma) was used. Internal standard working solution (100 μL), butylated hydroxy toluene (10 μL of a 50 mg/dL solution in ethanol) and potassium hydroxide (875 μL of 0.5 M in ethanol) were added in succession. Samples were perfused with argon, capped tightly and mixed (Glas-COL laboratory rotator, 3 hours at room temperature). Samples were neutralized to pH 7 by addition of 21 μL of phosphoric acid (85%) and 1 mL of water. Neutral pH was checked with litmus paper. Clear supernatant was obtained after centrifugation (3000 rpm, room temperature for 2 minutes).

### Solid Phase Extraction (SPE)

HyperSep C18 SPE (200 mg) tubes were conditioned with 1 mL of n-hexane:2-propanol (50:50 v/v) followed by 1 mL of methanol and then 2 mL of water using an SPE vacuum manifold. Sample supernatants were loaded to the SPE tube and allowed to flow under gravity. SPE tubes were washed with 2 X 2 mL of methanol: water (75:25 v/v) and then eluted using 2 mL of hexane:2-propanol (50:50 v/v) initially under gravity and then under negative pressure of the SPE manifold. The SPE eluate was evaporated under nitrogen at 37°C, reconstituted with 275 μL of methanol with vortexing followed by addition of 25 μL of water. Samples were loaded into 1.5 mL LC autosampler vials with 0.25 mL limited volume conical vial inserts and 50 μL was injected by the autosampler onto the LC- MS.

### Instrumentation

The LC-MS was a model LC 2010A (Shimadzu Scientific Instruments, Columbia, MD) equipped with two LC-10ADvp pumps, a low volume high pressure mixer, a SIL-HT autosampler, a CTO-10AC column oven, a SPD-M10A photo-diode array (PDA) Detector, a LCMS-2010A mass spectrometer with APCI interface, drying gas and computer system with LabSolutions software.

A flow control valve (FCV) was installed between the PDA and MS to divert the system flow away from the MS detector while allowing continued PDA monitoring. The PDA range was 190–250 nm with the primary analysis channel set at 204 nm for cholesterol and stigmasterol detection.

The MS parameters were optimized for the detection of 24(*S*)-hydroxycholesterol with the follow settings: interface: APCI, ion mode: positive, detector voltage: 1.5 kV, APCI Interface temperature: 400°C, CDL temperature: 230°C, nebulizing gas flow: 2.5 L/min, heat block temperature: 200°C and sampling: 1.5625 Hz (time constant: 0.640 sec). Selected ion-monitoring (SIM) time segments were employed to maximize the sensitivity of the MS detector as each cluster of chromatographic peaks eluted from the column.

### Chromatographic Conditions

A reverse phase C-18 Supelcosil LC-18-S, 25 cm x 4.6 mm, 5 μm column (Sigma Aldrich, St. Louis, MO) was used to achieve chromatographic separation. The autosampler and column oven were maintained at 10°C and 30°C, respectively. The mobile phase consisted of Solvent A: 100% methanol containing 0.1% formic acid and Solvent B: methanol: water (50:50 v/v) containing 0.1% formic acid. The gradient program was isocratic 80% A for 15 minutes, followed by a linear curve to 100% A from 15–20 minutes with the remainder of the run at 100% A. The flow rate was constant at 0.6 mL/min and the total run time was 45 minutes per sample.

Because cholesterol is present in high concentrations in plasma, a flow control valve (FCV) was positioned in-line between the diode array detector and the MS detector. The FCV diverted the cholesterol peak away from the MS detector between 32.5 and 36 minutes to prevent overloading of the ion source and MS detector. A re-equilibration phase between 38 and 45 minutes returned the system to the original conditions.

### Method Validation

Method validation was performed according United States Food and Drug Administration (FDA) guidelines for bioanalytical method validation [27].

Standard state-of-the art methods for calibration, evaluating extraction efficiency and matrix effects, precision and accuracy, lower limit of quantitation and sensitivity were used. These are described in S1 File which provides an expanded description of experimental procedures.

### External Reference Materials

Oxysterol and total cholesterol external reference materials were from “Survey for Sterols and Oxysterols 1/14” samples A, B, C and D from Referenzinstitut für Bioanalytik (Bonn, Germany). VitD3 proficiency was evaluated using standard reference material (SRM) 972a from the National Institute of Standards and Technology (NIST, Gaithersburg, MD).

### Analyte Stability Studies

Autooxidation during sample processing can cause loss of analyte oxysterols and produce oxysterol artifacts. Details of the stability testing procedures are provided in Supplementary Methods.

### UPLC-MS/MS Assay for Multiple Vitamin D Metabolites

Plasma 25-OH-Vitamin D3 levels for 78 randomly selected samples were analyzed in blind fashion and compared against values determined previously by UPLC-MS/MS [28]. For UPLC-MS/MS, 200 μl sample of plasma was vortex-mixed with 20 μl working internal standard solution (containing 20 ng/mL *d*6-25-OH VD_2_, 20 ng/mL *d*6-25-OH VD_3_, and 2.0 ng/mL *d*6-1α, 25-(OH)_2_-VD_3_) and allowed to equilibrate. After protein precipitation with methanol/acetonitrile, the supernatant was dried under nitrogen at room temperature. Selective derivatization of vitamin D analogs was accomplished with 4-phenyl-1, 2, 4-triazoline-3, 5-dione (PTAD) for 2 hours at room temperature followed by the addition of water to scavenge unreacted PTAD. The derivatization product was subject to an optimized, selective solid-phase extraction. After washes, the analytes were eluted. The eluate was evaporated, reconstituted in 100 μl of mobile phase, and 8 μl of the reconstituted eluate was injected into the UPLC-MS/MS system.

### Application to Human Studies

Human EDTA plasma samples (*n* = 220) were analyzed. Plasma was separated from EDTA blood collection tubes within 2 hours of the blood draw. Plasma was frozen in aliquots at -80°C promptly after separation. Blood samples were collected during the period March 2009 to August 2009 and analyzed in the second quarter of 2013. Samples included well-characterized MSC patients from a prospective study of clinical, environmental and genetic factors in MSC and healthy controls of similar age and sex distribution. All patients provided written informed consent. The study protocol was approved by the University at Buffalo Health Sciences Institutional Review Board.

Samples were analyzed as a continuous set of 22 consecutive batches of 10 samples. System suitability test mixtures, reagent and sample blanks, and quality control samples were included in every run.

For statistical analyses, study subjects were grouped into HC, MSC, and OND groups. The Kruskal-Wallis non-parametric ANOVA followed by post hoc Mann-Whitney tests were used to assess differences in oxysterol levels between the study groups.

## Results

### Chromatography and Detection

**Fig 1:**
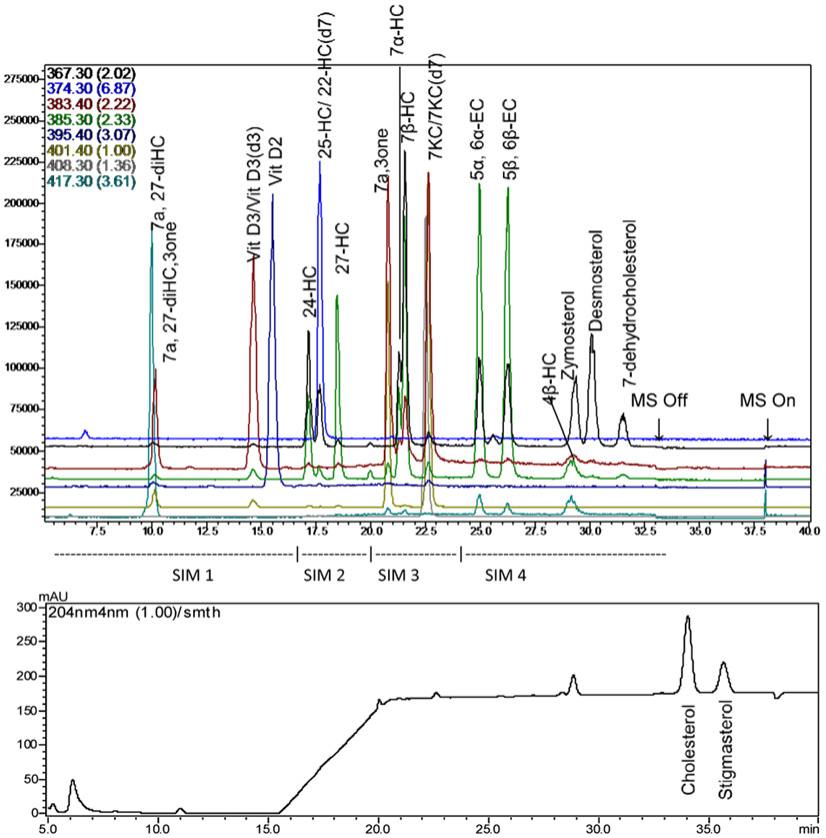
LC-MS-PDA chromatogram of standards. The top panel shows the chromatogram of each selected ion-monitoring (SIM) channel with each analyte identified at the peak apex. MS Off/MS On indicated the activation points for the flow control valve. The bottom panel shows the PDA Chromatogram at 204 nm.

The identification, acronyms, molecular weight, retention time, m/z ratio of the major APCI+ ion for each chromatographic peak and the division of the SIM time segments are summarized in Table 1. Fig 1 is a representative chromatogram of a system suitability test mixture containing 21 analytes including internal standards. The SIM time window segments are identified and the time of the diversion of the LC system flow away from the MS detector by the FCV is indicated. The mobile phase gradient is apparent in the baseline of the simultaneous PDA chromatogram at 204 nm (Fig 1, bottom panel). The peaks for total cholesterol and its internal standard stigmasterol are fully resolved. S1 Fig and S2 Fig show detailed total ion chromatograms and individual SIM time segments for the mixture of standards (S1 Fig) and a representative human plasma sample (S2 Fig). The mobile phase gradient was carefully optimized to resolve 24HC from 25HC and 7αHC from 7βHC. Limiting dilutions of mixed standards were analyzed to estimate the LOD and the LLOQ for each analyte. The chromatographic performance (resolution and asymmetry), LOD and LLOQ for each analyte are summarized in S1 Table.

**Table 1:** Analyte systematic name, acronym and the selected ion monitoring (SIM) segment windows used for APCI^+^-MS detection and the wavelengths for photodiode array detection.

Compound Name	Acronym	Target/IS	Monoisotopicmass	Retention Time (min)	APCI+ *m/z*
**Segment 1 (0–16.5 min)**					
7α,27-dihydroxycholesterol	7α,27diHC	Target	418.35	11.05	383.4
7α,27-dihydroxy-4-cholesten-3-one	7α,27diHC,3one	Target	416.33	10.25	417.3
25-hydroxy vitamin D3	VitD3	Target	400.33	14.85	383.4
25-hydroxyvitamin D3 (6,19,19-***d***3)	VitD3(*d*3)	IS	403.33	14.85	386.4
25-hydroxy vitamin D2	VitD2	Target	412.34	15.82	395.4
**Segment 2 (16.5–19 min)**					
24(S)-hydroxycholesterol	24HC	Target	402.35	17.25	367.3
25-hydroxycholesterol	25HC	Target	402.35	18.05	385.3
22-hydroxycholesterol (25,26,26,26,27,27,27-***d***7)	22HC(d7)	IS	409.39	18.06	374.3
(25R)26-hydroxycholesterol	27-HC	Target	402.35	18.52	385.3
**Segment 3 (19–23.5 min)**					
7α- cholestenone	7α,3one	Target	400.33	20.75	401.4
7α- hydroxycholesterol	7αHC	Target	402.35	21.25	367.3
7β- hydroxycholesterol	7βHC	Target	402.35	21.76	367.3
7-ketocholesterol	7KC	Target	400.33	22.54	401.4
7-ketocholesterol (25,26,26,26,27,27,27-***d***7)	7KC(d7)	IS	407.38	22.48	408.3
**Segment 4 (23.5–33.5 min)**					
5α,6α-epoxycholesterol	5α,6αEC	Target	402.35	25.05	385.3
5β,6β-epoxycholesterol	5β,6βEC	Target	402.35	26.47	385.3
4β-hydroxycholesterol	4βHC	Target	402.35	28.50	385.3
Zymosterol	Zymo	Target	384.34	29.25	367.3
Desmosterol	Desmo	Target	384.34	30.35	367.3
7-dehydrocholesterol	7-DHC	Target	384.34	33.10	367.3
**Photodiode Array**					**λ**_**max**_ **, nm**
Cholesterol	Chol	Target	386.36	34.60	206
Stigmasterol	Stig	IS	412.36	35.75	204

Peak retention time and mass to charge ratio (*m/z*) of the major fragment generated by each analyte are listed.

### Stability, Recovery and Matrix Effects

We assessed the formation of oxysterols from purified cholesterol samples processed with and without the addition of 0.5 M ethanolic KOH. No losses of cholesterol or the presence of any analytes was detected.

To determine the stability of endogenous oxysterols during sample preparation, a mixture of 21 analytes and internal standards was processed with and without alkaline hydrolysis.

Without base there was no significant difference in peak areas when compared to an unprocessed mixture of the 21 compounds after 3 hours, under argon, at room temperature or after 24 hours in the autosampler. However, two additional artifact peaks (indicated as A1 and A2 in S2 Fig) were present at approximately 19.0 min and 25.25 min in chromatograms of mixtures subject to 0.5 M ethanolic potassium hydroxide, regardless of reaction time (0 hours to 3 hours). In experiments with individual standards, the A1 peak at 19 min appeared upon the alkaline hydrolysis of either 5α6αEC or 5β6βEC and the A2 peak at 25.5 minutes appeared upon the alkaline hydrolysis of 7βHC (data not shown). The peak areas of 7βHC, 4βHC, 5α6αEC, 5β6βEC and 7DHC were reduced (Reduction >15%) concomitantly with the presence of the artifact peaks. These analytes were therefore considered unsuitable for further quantitative analysis. However they were maintained in the system suitability mixtures to monitor chromatographic resolution from target peaks. All other analytes maintained >95% of their original peak areas following alkaline hydrolysis and following 24 hour dwell time in the autosampler.

All analytes demonstrated acceptable recovery between 88–104% except for 7βHC, 4βHC, 5α6αEC, 5β6βEC and 7DHC which had been shown unstable to alkaline hydrolysis. Furthermore, 7α27diHC, desmosterol and zymosterol failed to achieve ≥85% recovery.

Six plasma samples spiked at low (LLOQ QC) and high (HQC) levels indicated good recoveries (91–103%) and apparent lack of matrix effects for the remaining 8 analytes; VitD2, VitD3, 24HC, 25HC, 27HC, 7αHC, 7KC and cholesterol. Because VitD2 levels in human plasma were well below the limit of detection in all 6 unspiked plasma samples, no further validation was attempted on VitD2.

Untreated, purified cholesterol samples contained trace amounts of a peak co-eluting with 7KC (~9000 area units). The cholesterol samples subject to specimen processing and alkaline hydrolysis showed a further slight elevation in the levels of this peak (to ~ 16,000 area units). Upon closer analysis, this peak was also apparent in chromatograms of pure, solvent based standards and blank matrix and we assumed it to be a contaminant. Mass spectral analysis of this peak indicated a major fragment ion at 338 *m/z* and a trace ion at 401.3 *m/z* providing a match with erucamide, a slip-agent used in laboratory plasticware.

### Assay Performance

A 5 or 6-point calibration with *R* ^*2*^ of greater than 0.99 was achieved using blank matrix calibrators for the seven compounds that passed the stability and recovery phases of method validation. S2 Table summarizes intra-assay accuracy, correlation coefficient, slope and intercept of each calibration over three consecutive days.

The accuracy and imprecision of the method was validated in separately prepared control materials in spiked blank matrix at three levels (LLOQ QC, MQC and HQC). All analytes met acceptability criteria for accuracy and imprecision (Table 2). The LLOQ QC sample 7KC level was set higher than other oxysterols based on its LOD/LLOQ estimates. Furthermore, the imprecision estimates for 7KC were the highest observed presumably due to the presence of the erucamide contaminant.

**Table 2:** Intra- and inter-assay imprecision for method validation samples.

	LLOQ QC				MQC				HQC			
Analyte	Mean ng/mL	Accuracy % Dev*	Intra-assay, %CV*	Inter-assay, %CV**	Mean ng/mL	Accuracy % Dev*	Intra-assay, %CV*	Inter-assay, %CV**	Mean ng/mL	Accuracy %Dev*	Intra-assay, %CV*	Inter-assay, %CV**
**VitD3**	3.2	7.8	9.2	9.8	25.7	-4.7	6.5	6.6	65.6	1.6	3.4	4.4
**VitD2**	3.4	14.5	7.2	8.3	27.4	-4.4	4.1	4.4	67.8	9.8	6.8	6.6
**24HC**	5.2	4.2	8.8	8.8	13.0	-6.6	8.9	7.3	43.5	6.4	8.3	8.2
**25HC**	4.9	3.4	6.7	6.0	12.4	-2.5	9.0	6.6	45.4	7.6	7.0	7.0
**27HC**	6.0	7.8	8.4	9.7	48.43	-3.1	5.7	4.6	173.9	11.0	8.6	9.6
**7αHC**	5.8	12.14	8.2	12.2	46.9	-4.7	7.2	5.6	169.1	9.7	9.7	8.7
**7-KC**	12.5	15.6	13.4	10.4	12.5	8.23	13.4	10.4	108.7	9.9	10.4	12.0
**Chol**	25	2.0	3.2	3.5	124	-1.6	3.9	3.4	300	-9.4	9.1	8.8

* *n* = 6 on each of three consecutive days, the maximum daily value is reported

** *n* = 18; 6 replicates on each of 3 consecutive days

### Application to Human Plasma Samples

**Fig 2:**
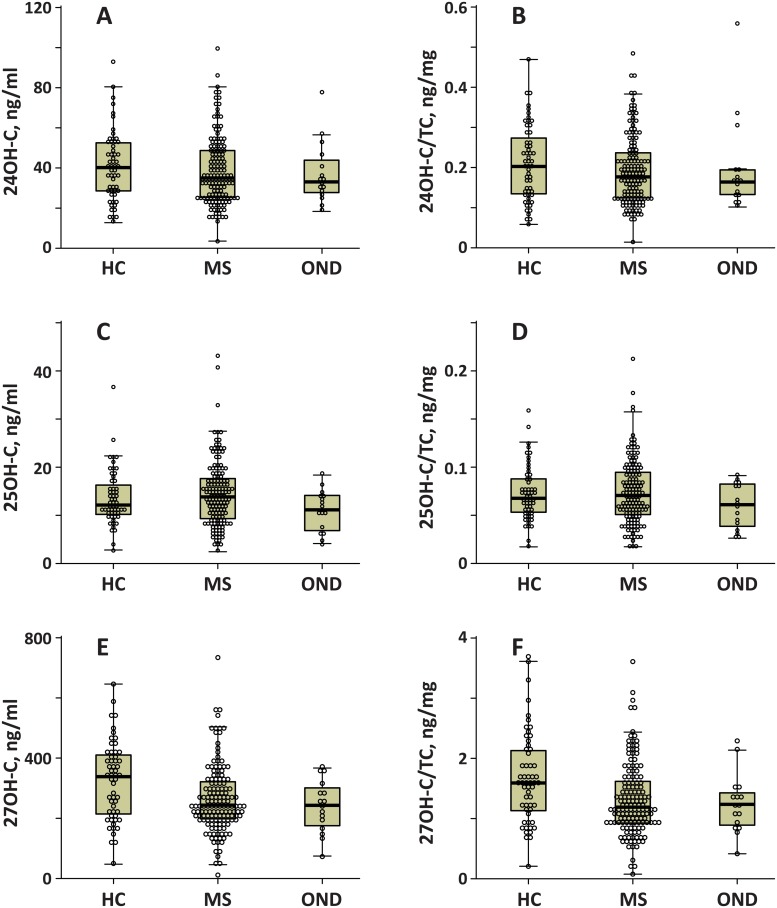
Side-chain oxygenated oxysterol levels in patient groups. Distribution of 24-hydroxy cholesterol (24HC; Fig 2A), 25-hydroxy cholesterol (25HC; Fig 2C) and 27-hydroxy cholesterol (27HC; Fig 2E) for healthy controls (HC), multiple sclerosis (MSC) and other neurological diseases (OND). The results in Fig 2B, 2D and 2F correspond to the oxysterols in Fig 2A, 2C and 2E, respectively, but are normalized to total cholesterol (TC) levels. The dot-plots of individual patients are superimposed on the box plots. The solid line in the box plots are the median, the box delineates upper and lower quartiles and the error bar represents the range. Panel E for 27HC and Panel F for 27HC-TC ratio contains the p-value from the Kruskal-Wallis test in the main body. The inset contains a bar graph and p-values from follow-up Mann-Whitney tests.

**Fig 3:**
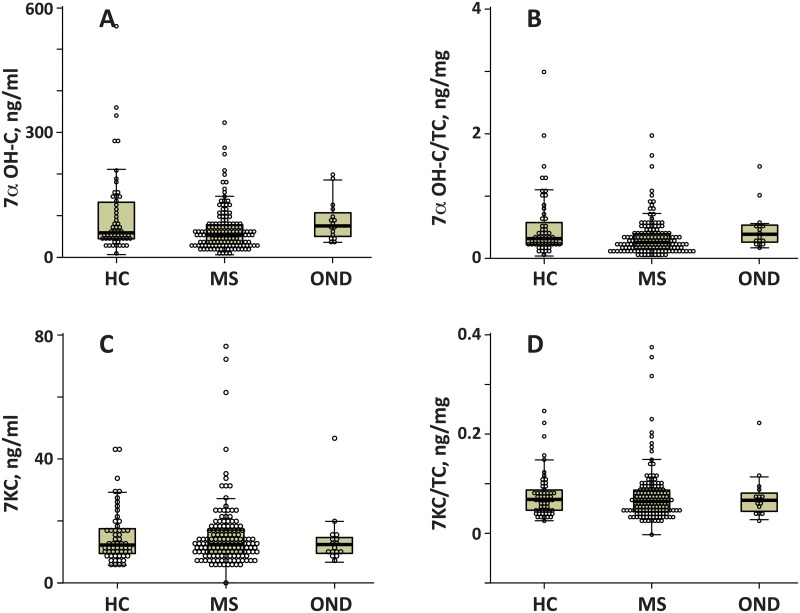
B-Ring oxygenated oxysterol and 25-hydroxy vitamin D3 levels in patient groups. Distribution of 7α hydroxy cholesterol (7αHC; Fig 3A) and 7 ketocholesterol (7KC; Fig 3C) and 25-hydroxy-vitamin D3 (25-OHVD_3_; Fig 3E) for healthy controls (HC), multiple sclerosis (MSC) and other neurological diseases (OND). The results in Fig 3B and 3C correspond to the oxysterols in Fig 3A and 3B, respectively, but are normalized to total cholesterol (TC) levels. The dot-plots for individual patients are superimposed on the box plots. The solid line in the box-plots are the median, the box delineates upper and lower quartiles and the error bar represents the range. Panel A for 7αHC and Panel B for 7αHC-TC ratio contains the p-value from the Kruskal-Wallis test in the main body. The inset contains a bar graph and p-values from follow-up Mann-Whitney tests. Panel E illustrates the agreement between 25-OHVD_3_ measured by our method on the y-axis compared to a validated LC-MS/MS method on the x-axis.

We deployed our method to analyze 220 plasma samples from 56 healthy controls (mean age ± SD = 46.7 ± 12 years, 54% female), 148 MSC patients (102 relapsing-remiting (RR-MSC) patients (age SD = 41.8 ± 9.6, 71% female), 40 secondary-progressive (SP-MSC) patients (age = 54.3 ± 8.5 years, 83% female), 6 primary-progressive (PP-MSC) patients (age = 50.8 ± 9.7 years, 83% female)), and 16 other neurological disorders (OND) patients (age = 49.8 ± 13, 69% female). The median disability on the expanded disability status scale (inter-quartile range) was 2.0 (1.0) for RR-MSC, 6.5 (1.5) for SP-MSC and 5.0 (3.3) for PP-MSC. Box plots illustrating the distribution of side chain oxygenated 24HC, 25HC and 27HC in control, MS and OND groups are shown in Fig 2. The corresponding distribution of cholesterol-normalized 24HC, 25HC and 27HC levels are also shown. The distribution of 7αHC and 7KC levels and the corresponding cholesterol normalized values are shown in Fig 3. Fig 3 also shows the distribution of 25 hydroxy vitamin D3 from our method. Significant changes were detected specifically in 7αHC, 27HC using Kruskal-Wallis tests examining all patient groups. Mean serum concentrations of 27HC were lower in MSC and OND groups relative to HC while 7αHC was significantly lower in the MSC when compared to HC or OND groups.

### Quality Control and Proficiency Testing

For routine imprecision monitoring, commercial, lyophilized clinical chemistry control materials and a pooled human plasma sample were analyzed repetitively to establish quality control ranges for longer-term quality control. S3 Table summarizes the imprecision performance of these materials across 18-months of analysis.

To further assess method accuracy we analyzed proficiency samples from Referenzinstitut für Bioanalytik for oxysterols and cholesterol. Table 3 summarizes our agreement with 8 to 11 participating laboratories determining oxysterols and cholesterol. Most analytes were in good agreement for the median of the sub-collective employing LC-MS methods. Cholesterol levels were in close agreement with all four samples and the %CVs among reporting labs were lowest for this analyte (A: 29% and B:33%). Our method most significantly deviated for 7αHC. However, across the reporting laboratories the %CVs for the 7-oxygenated cholesterols showed widest variability ranging from 47–129%, which is perhaps due to autooxidation during unrefrigerated shipping of samples by ordinary mail.

**Table 3:** Comparison of the assay to external reference materials.

Compound	Sample	Reference Mean (50pct)	Our Assay
**24HC ng/mL**	A	61 (57)	40
	B	38 (33)	26
**25HC, ng/mL**	A	15 (9)	11
	B	11 (8)	14
**27HC, ng/mL**	A	159 (124)	96
	B	163 (116)	116
**7αHC, ng/mL**	A	86 (79)	164
	B	83 (62)	197
**7-KC, ng/mL**	A	95 (31)	43
	B	146 (120)	179
**Chol, mg/mL**	A	169 (159)	159
	B	111 (117)	117
	C	147 (137)	140
	D	220 (203)	204
**VitD3, ng/mL**	1	28.8	30.2
	2	18.1	20.4
	3	19.8	20.8
	4	29.4	32.3

The oxysterol reference materials were from Referenzinstitut für Bioanalytik and the vitamin D3 reference material was from The National Institute of Standards and Technology. 50pct; 50th percentile of the corresponding LC-MS sub-collective

For 25-hydroxy vitamin D3, we used the NIST SRM 972a reference. There is good agreement with SRM 972a but an approximate 9% positive bias by our assay (Table 3). In order to further validate the simultaneous VitD3 analysis with our LC-MS oxysterol analysis method, a method comparison was conducted against a standalone FDA validated, reference UPLC-MS/MS method for vitamin D metabolites [22, 29]. The concentrations of 78 representative patient samples were analyzed by both methods. There was excellent concordance between the two methods. The mean VitD3 level from the LC-MS method was 29.55 ± 13.4 ng/ml compared to the 24.19 ± 11.32 ng/ml for the reference method. The bias calculated as average difference between the concentrations from two methods was 5.36 ng/mL and regression *R* ^2^ value was 0.943. The scatter plot and regression line for the method comparison between our method and LC-MS/MS is shown in Fig 3.

## Discussion

We have developed a LC-MS-PDA method for the simultaneous determination of 25-hydroxy vitamin D3, 5 major oxysterols and total cholesterol in human plasma. The strengths of this method are several. The ability to determine this panel of analytes simultaneously in small (200μL) volumes of plasma, with a simple sample preparation procedure without the need for derivatization make this method an efficient tool for the further investigation of these biomarkers in clinical samples. In other published methods for oxysterol measurement, the use of SPE has a key role in cholesterol separation from oxysterols. In the method presented here, SPE is employed to capture oxysterols and wash away more polar cholestenoic acids and steroids which elute near the solvent front (data not shown). Cholesterol is then separated in the analytical phase by diverting the LC flux to the UV detector. The chromatographic separation resolves oxysterol isomers 24HC and 25HC as well as 7αHC from 7βHC. This is important because these isomers have been associated with different metabolic pathways, can be present in plasma in significant amounts and even tandem mass spectrometry (MS/MS) cannot completely differentiate these isomers without chromatographic resolution. Using selected ion monitoring (SIM) and specific SIM time-segments the method can reliably detect VitD3 and validated oxysterol analytes with a sensitivity of 5 to 10 ng/mL. Using in-line photodiode array and a flow control valve we are able to achieve the simultaneous quantitation of total cholesterol with sensitivity to 1 μg/mL and linearity beyond 300 mg/dL. We have developed a suitable blank matrix and validated this method according to FDA guidelines.

We have analyzed several external reference materials and found our method to be in reasonable agreement. It is important to note that the sterol/oxysterol proficiency testing from Referenzinstitut für Bioanalytik is only conducting its second exercise. These samples were sent via regular mail and were unrefrigerated during shipping, which may have adversely affected oxysterol levels due to autooxidation. Undoubtedly, method accuracy and concordance among laboratories will improve as the proficiency exercises are refined.

Our method has significant advantages and some drawbacks in comparison to competing methods such as LC-MS/MS and GC-MS. Our LC-MS method is less technically demanding, more cost-effective and time-efficient for routine clinical analyses. However, it has lower sensitivity. Nonetheless, the limits of detection of our method easily enable detection of oxysterols in the ranges present in healthy and diseased clinical plasma samples by other techniques [23, 30, 31].

We invested effort in developing methods to obtain a blank matrix and evaluating the extent to which oxysterols were removed so that we could assess the potential for interference and the influence of other lipids on extraction efficiency and analyte recovery. The ubiquitous nature of cholesterol and its metabolites present well known challenges [32]. In some cases, blanks matrices might be synthesized or obtained from subjects with suppressed levels of the compound—this is not possible given the many essential physiological roles of cholesterol *in vivo*. We attempted to use fatty acid free bovine serum albumin as the protein component for a simulated matrix however we found that even this delipidated product still contained microgram quantities of cholesterol and measurable levels of oxysterols. Regardless, the included recovery studies in authentic plasma indicate that matrix effects are not present. Furthermore, subsequent studies have shown that solvent-based calibration solutions give nearly identical results compared with matrix-based calibrators, which further underscores the lack of matrix effects (data not shown). We have also invested time in applying this method to cerebrospinal fluid and brain tissue homogenates however further interference studies will be required to validate this method in these matrices.

We found analytical interference for 7KC from the common plastic slip-agent erucamide [33]. The *m*/*z* at 401.3 peak of erucamide coincides with a peak with the same *m/z* for 7KC. Our systematic assessment indicates this contaminant explains the higher limit of detection and imprecision for 7KC compared to other oxysterols. We were unable to entirely eliminate this contaminant despite attempts with specially washed pipet tips and extraction vials. Analytical samples that are close to the 7KC LLOQ (10.5 ng/mL) should be interpreted conservatively.

In conclusion, we have developed a robust and effective LC-MS-PDA profile for simultaneously measuring cholesterol, oxysterols and vitamin D3 in human plasma that may find numerous clinical and biomedical research applications in neurological and autoimmune disorders.

## Supporting Information

S1 Fig Detailed total ion chromatogram (TIC) and each corresponding selected ion monitoring (SIM) time segment (1–4) for a mixture of 20 analyte standards including internal standards. Peaks are labeled with the acronym as listed in Table 1.
(PPT)

S2 Fig Detailed total ion chromatogram (TIC) and each corresponding selected ion monitoring (SIM) time segment (1–4) for a representative plasma sample including internal standards. Peaks are labeled with the acronym as listed in Table 1. Peaks labeled A1 and A2 represent artifact peaks observed following sample processing.
(PPT)

S1 Table Chromatographic peak parameters and limits of detection for analytes. (DOC)

S2 Table Intra- day accuracy (range of % deviation from nominal value) of the calibration standards, regression coefficients values, slopes of the calibration curves and y- intercept values for 25-hydroxy-vitamin D3 (VitD3), five oxysterol standards and cholesterol on three different days. (DOC)

S3 Table Means, standard deviations and CV% for plasma QC sample stored at -80°C, and commercial, lyophilized, quality control materials. Plasma QC represents 88 replicates across 44 analytical batches and 17 months of analysis. DC-TROL I and II represent analyses across six separate lyophilized vials reconstituted from a single production lot number.
(DOC)

S1 File Expanded description of experimental procedures: (DOC)
