# Supplementary figures and images for: Simultaneous Determination of Oxysterols, Cholesterol and 25-Hydroxy-Vitamin D3 in Human Plasma by LC-UV-MS
Source: PLoS One. 2015 Apr 13;10(4):e0123771. doi: 10.1371/journal.pone.0123771 (PMC4395275; doi:10.1371/journal.pone.0123771)

## Slide 1
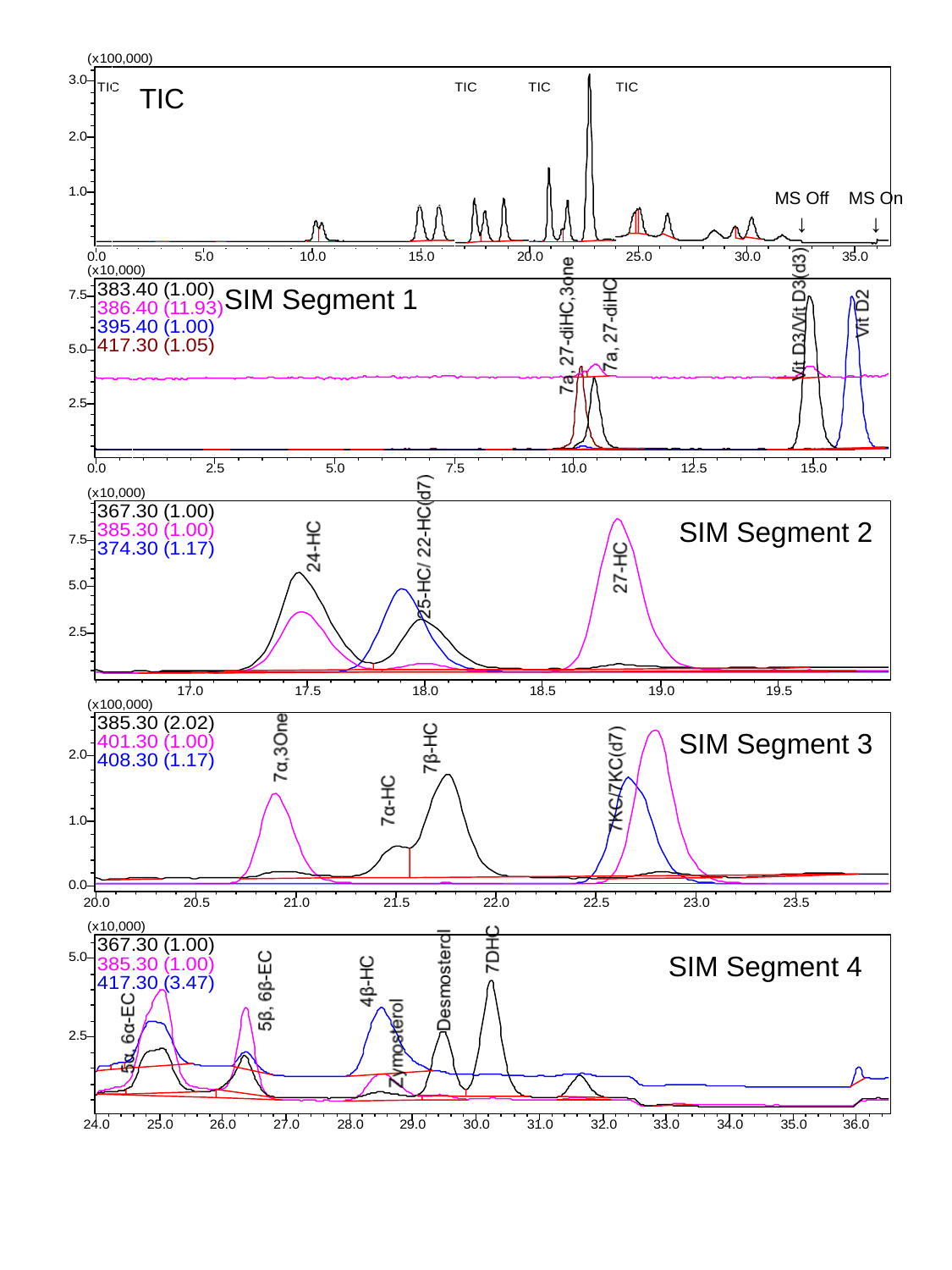

TIC
MS Off
↓
MS On
↓
SIM Segment 1
SIM Segment 2
SIM Segment 3
SIM Segment 4

Supplement: S1 Fig — Peaks are labeled with the acronym as listed in Table 1. (PPT) [file pone.0123771.s001.ppt]

## Slide 1
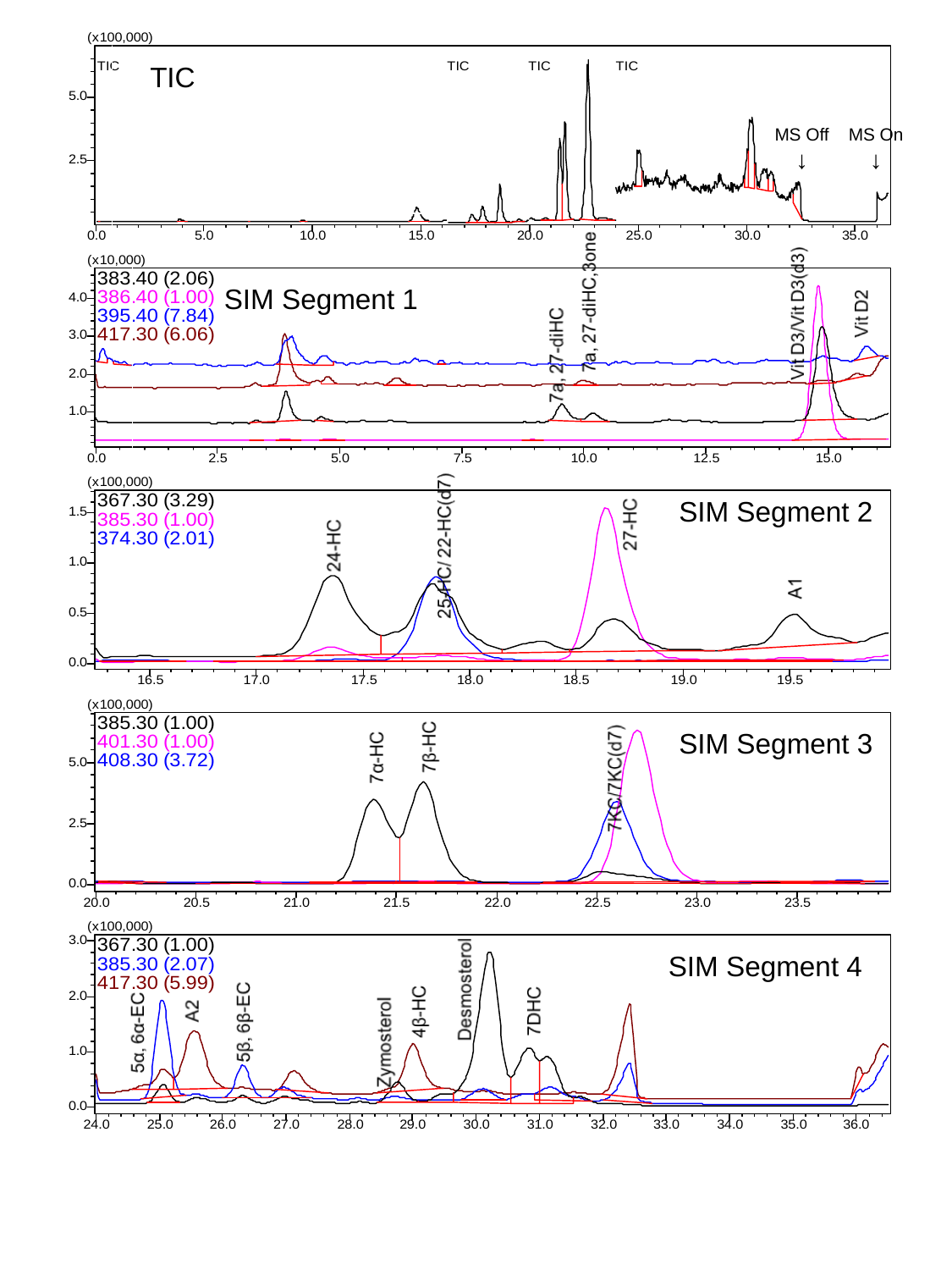

TIC
MS Off
↓
MS On
↓
SIM Segment 1
SIM Segment 2
SIM Segment 3
SIM Segment 4

Supplement: S2 Fig — Peaks are labeled with the acronym as listed in Table 1. Peaks labeled A1 and A2 represent artifact peaks observed following sample processing. (PPT) [file pone.0123771.s002.ppt]
