# Supplementary material for: Simultaneous Determination of Oxysterols, Cholesterol and 25-Hydroxy-Vitamin D3 in Human Plasma by LC-UV-MS
Source: PLoS One. 2015 Apr 13;10(4):e0123771. doi: 10.1371/journal.pone.0123771 (PMC4395275; doi:10.1371/journal.pone.0123771)
Supplement: S1 Table — (DOC) [file pone.0123771.s003.doc]

| **Compound Name** | **R_S_** | **A_S_** | **LOD**  **(ng/mL)** | **LLOQ**  **(ng/mL)** |
| --- | --- | --- | --- | --- |
| 7α,27-dihydroxycholesterol | >1.5 | 1.0 | 0.8 | 2.8 |
| 7α,27-dihydroxy-4-cholesten-3-one | >1.5 | 1.0 | 1.2 | 4.0 |
| 25-hydroxy vitamin D3 | >1.5 | 1.0 | 1.0 | 3.5 |
| 25-hydroxyvitamin D3 (6,19,19-d3) | >1.5 | 1.0 | NA | NA |
| 25-hydroxy Vitamin D2 | >1.5 | 1.0 | 1.1 | 3.5 |
| 24(S)-hydroxycholesterol | 1.1 | 1.0 | 1.2 | 4.0 |
| 25-hydroxycholesterol | 1.1 | 1.0 | 1.2 | 4.0 |
| 22-hydroxycholesterol (25,26,26,26,27,27,27-d7) | >1.5 | 1.0 | NA | NA |
| 27-hydroxycholesterol | >1.5 | 1.0 | 1.5 | 5.0 |
| 7α- cholestenone | 1.3 | 1.0 | 1.6 | 6.2 |
| 7α- hydroxycholesterol | 1.0 | 1.0 | 1.2 | 4.2 |
| 7β- hydroxycholesterol | 0.8 | 1.0 | 1.4 | 4.9 |
| 7-ketocholesterol | >1.5 | 1.0 | 3.2 | 10.5 |
| 7-ketocholesterol (25,26,26,26,27,27,27-d7) | >1.5 | 1.2 | NA | NA |
| 5α,6α-epoxycholesterol | >1.5 | 0.6 | 0.8 | 2.8 |
| 5β,6β-epoxycholesterol | >1.5 | 0.8 | 0.8 | 2.7 |
| 4β-hydroxycholesterol | 0.9 | 1.3 | 5.2 | 17.2 |
| Zymosterol | 1.0 | 1.6 | 4.4 | 13.7 |
| Desmosterol | 0.6 | 1.2 | 4.6 | 15.4 |
| 7-dehydrocholesterol | 0.6 | 1.3 | 7.5 | 26.0 |
| Cholesterol | >1.5 | 1.0 | 1050 | 4175 |
| Stigmasterol | >1.5 | 1.0 | NA | NA |

RS; Resolution calculated as Δt_R_/W_AVG_ for the closest adjacent peak at the same m/z ratio, AS; asymmetry calculated as tailing 50% of peak / leading 50% of peak, LOD; limit of detection (signal to noise ratio of 3:1), LLOQ; lower limit of quantification (signal to noise ratio of 10:1), NA; Not Analyzed
