# Supplementary material for: Simultaneous Determination of Oxysterols, Cholesterol and 25-Hydroxy-Vitamin D3 in Human Plasma by LC-UV-MS
Source: PLoS One. 2015 Apr 13;10(4):e0123771. doi: 10.1371/journal.pone.0123771 (PMC4395275; doi:10.1371/journal.pone.0123771)
Supplement: S2 Table — (DOC) [file pone.0123771.s004.doc]

| **Day** |  | **VitD3** | **24HC** | **25HC** | **27HC** | **7αHC** | **7 KC** | **Cholesterol** |
| --- | --- | --- | --- | --- | --- | --- | --- | --- |
| **1** | % Dev | -2.9-1.7 | -5.3-11.5 | -7.1-8.7 | -3.3-4.2 | -2.2-5.7 | -5.8-7.8 | -5.4-4.2 |
|  | *R^2^* | 0.9959 | 0.9979 | 0.9969 | 0.9994 | 0.9997 | 0.9998 | 0.9938 |
|  | Slope | 0.0126 | 0.0073 | 0.0037 | 0.0075 | 0.0084 | 0.0238 | 0.0601 |
|  | Intercept | 0.0164 | -0.0068 | -0.0012 | -0.0143 | -0.0327 | -0.0150 | 0.0203 |
| **2** | % Dev | -1.8-9.6 | -6.6-5.3 | -9.4-6.0 | -3.9 to 3.6 | -3.5-1.5 | -3.4-2.4 | -5.2-6.9 |
|  | *R^2^* | 0.9931 | 0.9984 | 0.9983 | 0.9992 | 0.9999 | 0.9999 | 0.9989 |
|  | Slope | 0.0128 | 0.0068 | 0.0035 | 0.0067 | 0.0075 | 0.0233 | 0.0591 |
|  | Intercept | 0.0160 | 0.0006 | 0.0012 | 0.0026 | 0.0023 | 0.0574 | 0.0199 |
| **3** | % Dev | -4.2-2.5 | -5.5-14.9 | 0.1-4.6 | -9.4-12.2 | -9.5-9.1 | -5.3-10.6* | -2.3-4.5 |
|  | *R^2^* | 0.9964 | 0.9980 | 1.0000 | 0.9952 | 0.9949 | 0.9980* | 0.9975 |
|  | Slope | 0.0137 | 0.0093 | 0.0048 | 0.0092 | 0.0096 | 0.00297* | 0.0613 |
|  | Intercept | 0.0130 | -0.0155 | -0.0054 | -0.0512 | -0.0416 | -0.0896* | 0.0211 |

* Calibration point L 3 was excluded from calculations since it was an aberrant value.
