# Supplementary material for: Simultaneous Determination of Oxysterols, Cholesterol and 25-Hydroxy-Vitamin D3 in Human Plasma by LC-UV-MS
Source: PLoS One. 2015 Apr 13;10(4):e0123771. doi: 10.1371/journal.pone.0123771 (PMC4395275; doi:10.1371/journal.pone.0123771)
Supplement: S3 Table — Plasma QC represents 88 replicates across 44 analytical batches and 17 months of analysis. DC-TROL I and II represent analyses across six separate lyophilized vials reconstituted from a single production lot number. (DOC) [file pone.0123771.s005.doc]

|  |  | **Vit D3** | **24HC** | **25HC** | **27HC** | **7αHC** | **7- KC** | **Cholesterol** |
| --- | --- | --- | --- | --- | --- | --- | --- | --- |
| **Plasma QC** | Mean | 42.35 | 95.38 | 70.70 | 193.39 | 43.57 | 100.40 | 185 |
| **(*n* = 44)** | SD | 3.35 | 3.90 | 10.98 | 13.99 | 7.12 | 17.54 | 13.6 |
|  | CV% | 7.9 | 4.1 | 15.5 | 7.2 | 16.4 | 17.5 | 7.3 |
| **DC-TROL I** | Mean | 24.25 | 40.57 | 65.59 | 357.18 | 1034.76 | 454.84 | 284.40 |
| **(*n* = 30)** | SD | 1.40 | 5.54 | 12.55 | 24.01 | 123.30 | 19.48 | 26.3 |
|  | CV% | 5.8 | 13.7 | 19.1 | 6.7 | 11.9 | 4.3 | 9.2 |
| **DC-TROL II** | Mean | 14.94 | 18.97 | 42.29 | 171.87 | 720.10 | 491.69 | 142.37 |
| **(*n* = 30)** | SD | 0.87 | 3.16 | 4.83 | 13.31 | 45.62 | 36.85 | 7.04 |
|  | CV% | 5.8 | 16.7 | 11.4 | 7.7 | 6.3 | 7.5 | 4.9 |
