# Supplementary material for: Simultaneous Determination of Oxysterols, Cholesterol and 25-Hydroxy-Vitamin D3 in Human Plasma by LC-UV-MS
Source: PLoS One. 2015 Apr 13;10(4):e0123771. doi: 10.1371/journal.pone.0123771 (PMC4395275; doi:10.1371/journal.pone.0123771)
Supplement: S1 File — (DOC) [file pone.0123771.s006.doc]

**Reagents and Standards.** Optima (LC-MS grade) water and organic solvents were from Fisher Scientific. All solvents were degassed by simultaneous sonication and filtration through 0.22 µm nylon membranes prior to use. Dextran-coated charcoal, butylated hydroxytoluene, high purity potassium hydroxide, phosphoric acid and cholesterol (>99%) were from Sigma-Aldrich (St. Louis, MO). Oxysterols, dihydroxycholesterols (7α, 27 dihydroxycholesterol), epoxycholesterols (5α,6α-epoxycholesterol and 5β,6β-epoxycholesterol), cholestenones (7α-hydroxycholestenone and 7α, 27 dihydroxycholestenone) cholesterol precursors (zymosterol, desmosterol and 7-dehydrocholesterol) and deuterated oxysterol internal standards were from Avanti Polar Lipids (Alabaster, AL. USA). Cholesterol, stigmasterol, vitamin D precursors (25-hydroxy vitamin D2 and 25-hydroxy vitamin D3) and deuterated 25-hydroxy vitamin D3 were obtained from Sigma-Aldrich (St. Louis MO. USA). Pre-purified nitrogen and argon gases (>99.99%) were from Jackson Welding Supply (Buffalo, NY. USA).

**Blank Matrix Preparation.** Lipid stripped human plasma, ostensibly free of oxysterols, vitamin D and cholesterol, was prepared by a modification of the methods of Cham and Cao .

Pooled human plasma was mixed with two volumes of butanol: diethyl ether (40:60 v/v), perfused with argon, vortexed and rotated at room temperature (30 minutes at 30 rpm). The aqueous phase was separated by centrifugation (3000 *g* for 5 minutes at 4°C) and removed by aspiration.

The partially stripped plasma was then treated with 2.5% (w/v) dextran-coated charcoal, rotated overnight at room temperature. The charcoal was separated by centrifugation (3000 *g* for 5 minutes at 4° C). The supernatant (4 mL portions) was passed through a HyperSep C-18 solid phase extraction tube (500 mg bed volume) and sterile filtered (Corning 0.2 µm PES membrane filter).

The quality of the blank matrix was assessed by analysis for cholesterol, cholesteryl esters and oxysterols before and after delipidation. Cholesterol and cholesteryl analyses were done using HPLC according to Vercaemst and oxysterol analysis was carried out using the LC-MS method described below.

**Preparation of Calibrators, Internal Standards and Quality Control Samples.** Stock cholesterol and oxysterol standards were prepared by dissolving the powdered form of the standard in degassed, LC-MS grade methanol at a concentration of 1.0 -2.0 mg/mL. Stock VitD2 and D3 standards were generated using a molar extinction coefficient of 18,300 AU·M^–1^·L^–1^ at 265 nm in ethanol . Prepared stock standards were perfused with argon and stored at -20°C. Calibrators were prepared by spiking precise amounts of each stock standard into lipid stripped, blank plasma matrix to achieve final calibrator concentrations. The organic solvent content of all matrix calibrators was <5%.

An internal standard (IS) working solution consisted of 150 ng/mL each of 22-HC(*d*7), 7KC(*d*7) and VitD3(*d*3) as well as 250 μg/mL of stigmasterol. 22HC(*d*7) served as internal standard for 24HC, 25HC and 27HC. VitD3(*d*3) served as the internal standard for VitD2 and VitD3. 7KC(d7) served as internal standard for 7αHC and 7KC. Stigmasterol served as the internal standard for cholesterol.

Samples of known concentration for validation studies were prepared by spiking known amounts of standards into blank matrix. Validation samples were prepared at three levels; at the lower level of quantification (LLOQ QC), a mid-range (MQC) and a high level (HQC). Validation samples were prepared from standard stock solutions prepared separately from the calibrator standards. For routine QC we used DC-TROL multi-analyte, lyophilized control materials from Sekisui Diagnostics (Lexington, MA). Additional QC material for long-term storage studies consisted of in-house human plasma pools stored in aliquots at -80°C.

**Calibration.** Calibration curves were derived from plots of the ratio of analyte area to internal standard area versus calibrator concentration. Calibration points were obtained using calibrators at six levels (L1 to L6). A calibration curve was accepted if calculated concentrations of the L2-L6 calibration standards from the linear regression line were within ±15% of the nominal value and the L1 (LLOQ standard) was within ±20%. At least 80% of standards (5 of 6 calibrators) were required to meet these criteria.

**Extraction Recovery and Matrix Effects.** Extraction efficiency for each analyte and internal standard was assessed by preparing identical concentrations of analyte in both blank matrix and in pure solvent. Triplicate blank matrix based samples were subjected to sample preparation while triplicate solvent based analytes were analyzed directly. Recovery was calculated as the percentage of extracted, matrix-based samples relative to the solvent-based samples. Recoveries greater than 85% were considered acceptable. Sample based recovery and matrix effects were assessed by spiking known amounts of methanol solutions of analytes into each of six separate human EDTA plasma samples. Samples were spiked prior to sample preparation or sample extracts were spiked immediately prior to analysis. Spike solutions were consistent with the LLOQ QC and HQC levels and the solvent content of spiked plasma samples was < 5% of the total sample volume. Recovery was calculated by the method of standard additions and a %recovery between 85 and 115% (± 15%) for all 6 plasma matrices was considered indicative of a lack of matrix effects.

**Precision and Accuracy.** Intra-assay precision and accuracy was determined by analyzing six replicates each of LLOQ QC, MQC and HQC on three consecutive days. Accuracy was expressed as percent deviation from the nominal concentration and precision was expressed in terms of percent coefficient of variation (%CV). Inter-assay precision and accuracy were determined by pooling the replicate intra-assay analysis on three different days. Accuracy was considered acceptable if the interpolated concentration at each QC level was within 15% of nominal value, except for the LLOQ, where it could be within 20% of nominal. The acceptance criteria for precision was 15% CV, except for the LLOQ, which should not exceed 20% CV.

**Lower Limit of Quantification (LLOQ) and Sensitivity.** Guided by preliminary calibration curves, we estimated the signal to noise ratio at the lower limits of detection. Replicate analysis of six samples was carried out and the relative peak height of the analyte was compared to the relative height between the minimum and maximum points on the chromatogram baseline (noise). The limit of detection (LOD) was determined as the signal/ noise ratio greater than 3:1; the lower limit of quantification (LLOQ) was determined as the signal/ noise ratio greater than 10:1.

**Analyte Stability Studies.** Autooxidation during sample processing can cause loss of analyte oxysterols and produce oxysterol artifacts. Commercial grade cholesterol (>98%) was re-purified using reverse phase HPLC to remove any traces of oxysterols. A methanol solution of purified cholesterol standard (300 mg/dL) was subjected to the sample preparation steps with and without addition of potassium hydroxide (0.5 M) in ethanol. The samples were analyzed by HPLC-MS for the presence of oxysterols before reaction, immediately after addition of base, and again after completion of 3 hours of alkaline hydrolysis reaction.

To test for the possible destruction of oxysterols compounds during sample processing, individual oxysterol standards as well as a mixture of the 21 oxysterols, vitamin D and sterol standards were subject to sample preparation steps with and without addition of potassium hydroxide (0.5 M) in ethanol. The samples were analyzed by LC-MS before reaction, immediately after addition of base, and after completion of 3 hours of alkaline hydrolysis reaction. Stability of prepared samples within the autosampler was estimated at the LLOQ QC and HQC levels. Six replicates of each sample were re-analyzed after 24 hours in the autosampler. A difference of ≤ 15% was considered acceptable.

**VIII. REFERENCES**

[1] B.E. Cham, B.R. Knowles, J Lipid Res 17 (1976) 176.

[2] Z. Cao, C. West, C.S. Norton-Wenzel, R. Rej, F.B. Davis, P.J. Davis, Endocr Res 34 (2009) 101.

[3] R. Vercaemst, A. Union, M. Rosseneu, I. De Craene, G. De Backer, M. Kornitzer, Atherosclerosis 78 (1989) 245.

[4] G.D. Carter, J.C. Jones, Annals of Clinical Biochemistry 46 (2009) 434.

[5] P. CampinsFalco, L. GalloMartinez, A. SevillanoCabeza, F. BoschReig, Analytical Letters 29 (1996) 2039.
